# Relation Between Metabolic Activity of the Broca Region and F-18 FDG Uptake in Vocal Cords

**DOI:** 10.4274/Mirt.182

**Published:** 2012-08-01

**Authors:** Mine Şencan Eren, Hatice Durak

**Affiliations:** 1 Dokuz Eylül University School of Medicine, Department of Nuclear Medicine, İzmir, Turkey

**Keywords:** Positron-emission tomography, vocal cords, broca area

## Abstract

**Objective:** We aimed to investigate if increased F-18 Fluoro Deoxyglucose (F-18 FDG) uptake observed in vocal cords (VC) of the patients on Positron Emission Tomography/Computarize Tomography (PET/CT) scans is connected to speaking of the patients or not. If so, we expected to detect an increased metabolic activity in Broca's area. In this study, we have retrospectively searched for a correlation between the activity in the Broca's area and vocal cords of patients who had undergone FDG PET for different indications.

**Material and Methods:** FDG PET/CT scans of 30 patients with (VC [+]) and 30 patients without (VC [-]) bilateral F-18 FDG uptake on their vocal cords were retrospectively evaluated. Brain quantification was carried out on NeuroQ software with 20 iterations using patients' transaxial brain cross sections. On the 20th-23rd-26th-29th cross sections, area/whole brain ratios of the right (R) and left (L) for Broca’s area were calculated. VC (+) and VC (-) patients' R and L Broca's areas were compared using Student's t-test.

**Results:** There was no significant difference between the Broca's areas of VC (+) and VC (-) patients. L Broca's areas of both VC (+) and VC (-) patients were more active than R Broca's areas (p<0.05). There was a negative correlation between VC (+) patients' SUVmax values in the vocal cords and the activity in their R Broca's region.

**Conclusion:** In our study, we did not find a significant difference between Broca's areas of VC (+) patients and VC (-) patients, so the activity in their vocal cords does not seem to be related to increased metabolic activity in Broca's areas. We have concluded that the vocal cord activity is not related to speaking of the patients. The activity in the vocal cord might be due to inflammation or, as in the eye muscles, may be associated with high metabolism in laryngeal muscles.

**Conflict of interest:**None declared.

## INTRODUCTION

F-18 Fluorodeoxyglucose (FDG) Positron Emission Tomography/Computed Tomography (PET/CT) is used in a wide range of oncological patients. It helps the clinician in diagnosis, staging, biopsy guidance, response to treatment and re-staging processes (1). In addition, PET/CT is also used for imaging brain metabolism in conditions like dementia, epilepsy and psychiatric disorders (2).

FDG is a glucose analogue and it enters into the brain cells via the neuronal membrane glucose transporter. Deoxyglucose is converted to deoxyglucose-6-phosphate in neurons and captured in the cell.

Glucose metabolism varies in different brain regions even in healthy subjects (2,3). Speaking is carried out with a coordinated work of pharynx, larynx and respiratory muscle groups and with the activation of primary motor cortex (4). Localization of speech and Broca's area in the brain was identified by receptor studies (5). It was shown that Wernicke's and Broca's areas in the brain show increased metabolism during speech (4). In a number of studies on aphasic patients, damage was identified in Broca's area, which is the primary motor cortex of speech (6). 

FDG uptake is also observed in vocal cords (VC) which may be secondary to malignancy but it may as well be physiological (7,8,9,10).

In our study, we tested the hypothesis that patients with symmetrical FDG uptake in VCs in PET/CT might have spoken during the imaging process and if they had, Broca's areas in the brain might have been activated secondary to speaking. We have investigated if patients with FDG uptake in their VCs have increased metabolic activity in Broca's areas.

## MATERIALS AND METHODS

Patients referred to our department between 2010 July and 2011 February for F-18 FDG PET/CT due to oncologic diseases were retrospectively evaluated. A total of 60 patients were selected as 30 patients with (VC [+]) and 30 patients without (VC [-]) bilateral F-18 FDG uptake on their vocal cords. Patients with a history of previous head and neck cancer, head and neck surgery, vocal cord paralysis, cerebrovascular disease (CVD), head and neck radiotherapy (RT), brain metastases and neurological disease were excluded. None of the patients had hoarseness. All the patients' fasting blood glucose (FBG) were measured after 6-8 hours of fasting. Patients’ FBG was ≤200 mg/dL. 

Patients rested in a quiet room after an intravenous (IV) catheter was placed. All patients were scanned about 60 minutes after the injection of 6.8 to 12.4 mCi F-18 FDG intravenously. Imaging was carried out using Phillips Gemini TOF PET/CT (Phillips Medical Systems Cleveland, Ohio, USA). First a low dose CT scan with 120 kV, 50-60 mA, 5 mm cross section was performed between vertex and 1/3 proximal femur. CT images were used for PET attenuation correction and anatomical localization purposes. Then, PET scan was performed for 1.5 minutes per bed position, from 1/3 proximal femur to vertex. 

Transaxial, coronal, sagittal and 3D fusion images of patients with and without attenuation correction were reviewed and bilateral VC (+) and VC (-) patients were identified. "Standard Uptake Value Maximum" (SUVmax) values were used to characterize the metabolic activity in vocal cords of VC (+) patients. Cross-sections were set parallel to orbitomeatal axis. 

After the cross-sections were created, they were processed with 20 iterations using NeuroQ (Cardinal Healthcare) software.After processing, average pixel values in standardized regions of interest (ROI) in Broca's areas in 20^th^, 23^rd^, 26^th^ and 29^th^ cross-sections were automatically calculated. Area/whole brain ratios of Broca's areas were obtained as left (L) and right (R) Broca 20, Broca 23, Broca 26 and Broca 29. Ratios to whole brain in each ROI were compared to the normal values in the database. L Broca/R Broca ratios were also calculated.

The data were evaluated with the SPSS 16.0 statistical software. The correlation between SUVmax values and area/whole brain ratios of Broca's areas in VC (+) patients were evaluated with Pearson correlation test. The difference between area/whole brain ratios of Broca's areas in VC (+) and VC (-) patients and the difference between the patients' area/whole brain ratios of right and left Broca's areas were evaluated with Student's t-test. p<0.05 was accepted as statistically significant.

## RESULTS

Fourteen of VC (+) patients were female, 16 were male and average age was 53.3±16.8. Average FBG of VC (+) patients was 104.2±18.9 mg/dL. Average dose of injected F-18 FDG was 9.7±0.7 mCi. F-18 FDG PET/CT was performed in 7 patients for diagnosis (3 mediastinal mass, 2 mesothelioma, 1 solitary pulmonary nodule, 1 unknown primary tumor), 3 patients for biopsy guidance (3 lung masses), 4 patients for staging (1 malignant melanoma, 1 lung squamous cell carcinoma, 1 colon cancer, 1 Hodgkin's lymphoma), 9 patients for evaluation of response to treatment (4 lung cancer, 2 non-Hodgkin's lymphoma, 1 colon cancer, 1 Hodgkin's lymphoma, 1 Ewing's tumor) and 7 patients for re-staging (2 ovarian cancer, 2 breast cancer, 1 gastrointestinal stromal tumor, 1 cervical cancer, 1 colon cancer). 

Average SUVmax value in vocal cords of VC (+) patients was 4.8±2.2 (2.6-12.3). Laryngeal examination of the patients with SUVmax values greater than 9.9 did not reveal any pathology. Laryngeal examination was not carried out for other patients.

Twelve of VC (-) patients were female, 18 were male and average of age was 58.5±12.1. Average FBG of VC (-) patients was 104.3±28.7 mg/dL. Average dose of injected F-18 FDG was 9.9±0.7 mCi. F-18 FDG PET/CT was performed in 5 patients for diagnosis (2 solitary pulmonary nodules, 1 mediastinal mass, 1 mesothelioma, 1 malignant melanoma), 3 patients for biopsy guidance (5 lung mass), 4 patients for staging (1 breast cancer, 1 colon cancer, 1 non-Hodgkin's lymphoma), 9 patients for evaluation of response to treatment (2 lung cancer, 2 gastric cancer, 2 non-Hodgkin's lymphoma, 2 Hodgkin's Lymphoma, 1 Langerhans' cell histiocytosis) and 8 patients for re-staging (2 breast cancer, 1 stomach cancer, 1 malignant melanoma, 1 squamous cell skin cancer, 1 pancreas cancer, 1 cervical cancer, 1 colon cancer). 

Average whole brain ratios of L and R Broca 20-23-26-29 areas in VC (+) and VC (-) patients were given in Table 1. No statistically significant difference was found between Broca/Whole brain ratios of vocal cord active and inactive patients.

Comparison of L Broca 20-23-26-29 and R Broca 20-23-26-29 areas in VC (+) and VC (-) patients are given in Table 2. There is a significant difference between L Broca 20-23-26-29 areas and R Broca 20-23-26-29 areas in VC (+) patients and between L Broca 20-23-26-29 areas and R Broca 20-23-26-29 areas in VC (-) patients. Left Broca areas had higher ratios compared to right (p<0.05). 

L Broca/R Broca ratios of VC (+) and VC (-) patients are given in Table 3 and no difference was observed between them. 

We could not find a strong correlation between SUVmax values of VC (+) patients and Broca's areas. We have found a very weak correlation with L Broca 20-23-26, R Broca 20-26-29 and a negative correlation with L Broca 29, R Broca 23. The correlation data between Broca areas and SUVmax values are given in Table 4. 

## DISCUSSION

PET and Functional Magnetic Resonance (fMRI) are two methods which provide clear appearances of active cortical locations during mental tasks. Both methods operate on the principle that an increase in blood supply is required for supplying oxygen in order to preserve increase in neural activity while a particular region of brain performs a particular task. In general, this condition occurs several seconds after neural activity starts and therefore, those techniques most likely offers indirect appearance of timing and sequence of cortical events (11). 

Studying meta-analysis of neuroimaging studies, Indefrey and Levelt plotted several regions related with word generation. They suggested that word selection is related with left middle temporal gyrus and phonological awareness is related with right supplementary motor cortex (SMC), left anterior insula and left posterior superior and middle temporal gyrus (Wernicke’s area). It was suggested that Broca’s area (left posterior inferior frontal gyrus) is significantly related with process of syllabication during word generation. It is not known whether sign generation and voice generation are equally related with those neural regions (12).

Neurosurgeon Wilder Penfield was the first scientist to experimentally prove the relationship between Broca’s area and speech generation. He reported in dozens of cases that in recovered patients who underwent neurosurgical operation due to treatment-resistant epilepsy, inferior frontal gyrus stimulations – there was slight inter-subject variations- via electrical stimulation of frontal lobe had the influence to stop ongoing speech. The coincidence between focus of Penfield’s effect and location of Broca’s area was a very convincing argument favoring motor role of this region (13).

In a study conducted on dead subjects and healthy (with regards to hearing ability) subjects, he revealed that left inferior gyrus was equally used by deaf and healthy subjects during voice and sign generation (14).

As it is estimated, both voice generation and sign generation are related with left inferior frontal gyrus and particularly with BA 45, the anterior section of Broca’s area. Equal activation of Broca’s area for both sign and word indicates that there is a modality-independent role of this region in language generation, consistent with findings of previous investigations. Accordingly, function of Broca’s area has a strong relationship with verbal – acoustic phonological features of verbal language (14). 

In another study, presence of activation in left inferior frontal gyrus during motion of talking was demonstrated using fMRI (15).In another study, F-18 FDG uptake was examined in vocal cords of patients speaking and not speaking before and after administration of F-18 FDG. Kostakoglu et al. observed uptake related with laryngeal muscle activity in patients talking before, during and after FDG administration, while they could not observe this activity in laryngeal region in non-talking patients (16).

All of those studies demonstrate that Broca’s area is activated during speech. In the current study, we examined increased metabolic activity in Broca’s area in order to prove whether FDG uptake or in other words increased metabolic activity in vocal cords is related with the speaking of the patients during examination. 

However, we could not find a significant correlation between FDG uptake in vocal cords and metabolic activity in Broca’s areas. Moreover, no significant difference was found between Broca’s areas of VC (+) patients and VC (-) patients.However, there was a significant difference between left Broca’s areas and right Broca’s areas of VC (+) patients (inter-group comparison) and also between left Broca’s areas and right Broca’s areas of VC (-) patients (inter-group comparison), left Broca’s areas having higher values than right Broca’s areas in all patients (p<0.03). 

No comparison was made with other publications since there is no similar study in the literature. It is concluded that activity in vocal cords is not related with increased metabolic activity in Broca’s areas and thus, it does not result from speaking of the patient, since no significant difference was found between Broca’s areas of VC (+) and VC (-) patients. It was considered that the metabolic activity in vocal cords may be secondary to inflammatory processes or it may be related with continuous high metabolism, similar to that of eye muscles. 

In current study, patients had PET/CT studies for oncologic purposes. Cranial PET/CT was not performed. Functional Brain PET studies need special patient preparation and acquisition techniques/parameters. On the other hand, it is also very well known that standard oncological FDG PET/CT imaging could not meet most of these requirement. Therefore, sensitivity of the study may be low considering the possibility of data loss.

## CONCLUSION

We think that the activity in vocal cords is not related to the vocal activity of the patient during the scan. The increased metabolic activity in vocal cords might be due to inflammation or related to a high metabolism in the laryngeal muscles. 

## Figures and Tables

**Table 1:**
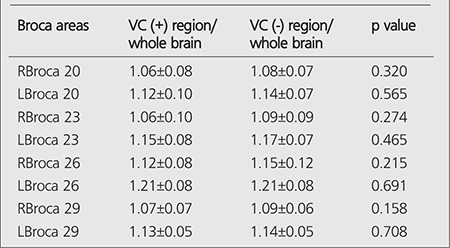
Average Broca's area/whole brain ratios of VC (+) and VC (-) patients and t-test results

**Table 2:**
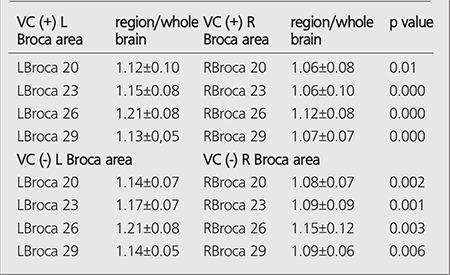
Comparison of left and right Broca regions of VC (+) and VC (-) patients and t-test results

**Table 3:**
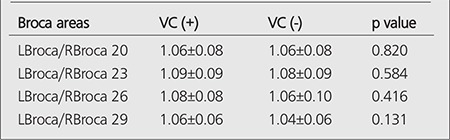
t-test results of VC (+) and VC (-) patients regarding L Broca/R Broca ratios

**Table 4:**
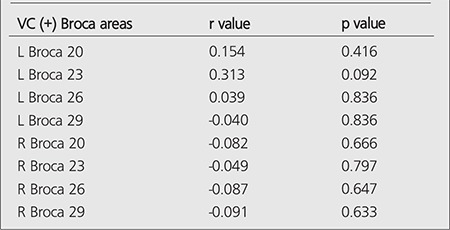
Correlation values of VC (+) patients regarding SUVmax values and Broca’s areas
